# Diethyl 4-[2-(4-meth­oxy­phen­yl)-1*H*-pyrazol-3-yl]-2,6-dimethyl-1,4-dihydro­pyridine-3,5-dicarboxyl­ate

**DOI:** 10.1107/S1600536811017600

**Published:** 2011-05-14

**Authors:** Hoong-Kun Fun, Madhukar Hemamalini, A. M. Vijesh, A. M. Isloor, Shridhar Malladi

**Affiliations:** a X-ray Crystallography Unit, School of Physics, Universiti Sains Malaysia, 11800 USM, Penang, Malaysia; b Department of Chemistry, National Institute of Technology, Karnataka, Surathkal, Mangalore 575 025, India

## Abstract

In the title compound, C_23_H_27_N_3_O_5_, the pyrazole ring is inclined at dihedral angles of 38.16 (6) and 80.80 (6)°, respectively, to the least-squares planes of the benzene and dihydro­pyridine rings. In the crystal, adjacent mol­ecules are linked *via* a pair of N—H⋯N hydrogen bonds, forming an inversion dimer. The dimers are stacked in a column along the *a* axis through N—H⋯O hydrogen bonds. Intra- and inter­molecular C—H⋯N and C—H⋯O hydrogen bonds are also observed.

## Related literature

For applications of pyridine derivatives, see: Surendra Kumar *et al.* (2011 ▶); Swarnalatha *et al.* (2011 ▶). For the stability of the temperature controller used in the data collection, see: Cosier & Glazer (1986 ▶).
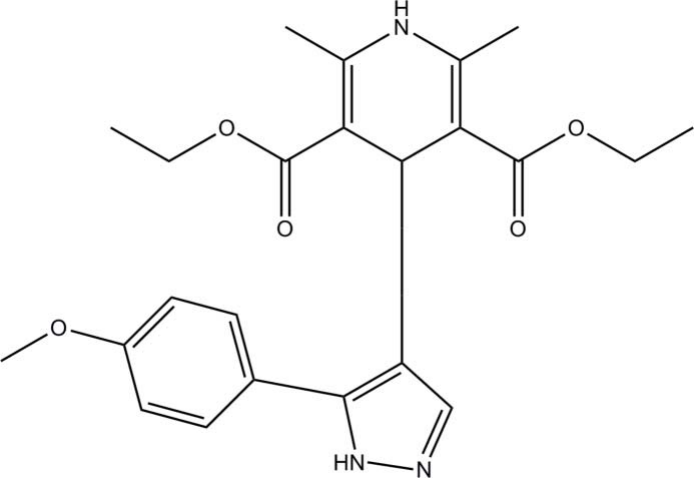


## Experimental

### 

#### Crystal data

C_23_H_27_N_3_O_5_
*M* *_r_* = 425.48
Triclinic, 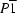

*a* = 8.5800 (1) Å
*b* = 11.1286 (1) Å
*c* = 11.4996 (1) Å
α = 94.425 (1)°
β = 99.191 (1)°
γ = 92.992 (1)°
*V* = 1078.37 (2) Å^3^
*Z* = 2
Mo *K*α radiation
μ = 0.09 mm^−1^
*T* = 100 K
0.39 × 0.23 × 0.22 mm

#### Data collection

Bruker SMART APEXII CCD area-detector diffractometer
Absorption correction: multi-scan (*SADABS*; Bruker, 2009 ▶) *T*_min_ = 0.965, *T*_max_ = 0.980
28279 measured reflections
7437 independent reflections
5974 reflections with *I* > 2σ(*I*)
*R*_int_ = 0.022

#### Refinement

*R*[*F* ^2^ > 2σ(*F* ^2^)] = 0.046
*wR*(*F* ^2^) = 0.134
*S* = 1.05
7437 reflections
293 parameters
H atoms treated by a mixture of independent and constrained refinement
Δρ_max_ = 0.51 e Å^−3^
Δρ_min_ = −0.29 e Å^−3^

### 

Data collection: *APEX2* (Bruker, 2009 ▶); cell refinement: *SAINT* (Bruker, 2009 ▶); data reduction: *SAINT*; program(s) used to solve structure: *SHELXTL* (Sheldrick, 2008 ▶); program(s) used to refine structure: *SHELXTL*; molecular graphics: *SHELXTL*; software used to prepare material for publication: *SHELXTL* and *PLATON* (Spek, 2009 ▶).

## Supplementary Material

Crystal structure: contains datablocks global, I. DOI: 10.1107/S1600536811017600/is2709sup1.cif

Structure factors: contains datablocks I. DOI: 10.1107/S1600536811017600/is2709Isup2.hkl

Supplementary material file. DOI: 10.1107/S1600536811017600/is2709Isup3.cml

Additional supplementary materials:  crystallographic information; 3D view; checkCIF report

## Figures and Tables

**Table 1:** Hydrogen-bond geometry (Å, °)

*D*—H⋯*A*	*D*—H	H⋯*A*	*D*⋯*A*	*D*—H⋯*A*
N2—H1*N*2⋯O1^i^	0.906 (17)	1.981 (17)	2.8858 (12)	176.3 (14)
N1—H1*N*1⋯N3^ii^	0.880 (17)	2.173 (17)	2.9969 (13)	155.7 (16)
C6—H6*B*⋯N3^iii^	0.98	2.50	3.4076 (15)	154
C7—H7*A*⋯O3^iv^	0.98	2.59	3.5561 (15)	167
C14—H14*A*⋯N1	0.95	2.60	3.2484 (14)	126
C18—H18*A*⋯O1^v^	0.95	2.48	3.1594 (14)	128
C22—H22*A*⋯O3	0.95	2.28	3.2210 (15)	170

Symmetry codes: (i) 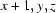; (ii) 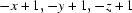; (iii) 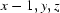; (iv) 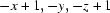; (v) 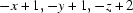.

**Fig. 1.**
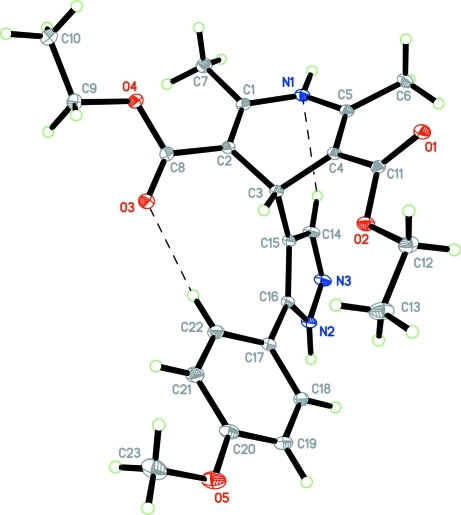
The molecular structure of the title compound, showing 30% probability displacement ellipsoids and the atom-numbering scheme. C—H···O and C—H···N hydrogen bonds are shown by dashed lines.

**Fig. 2.**
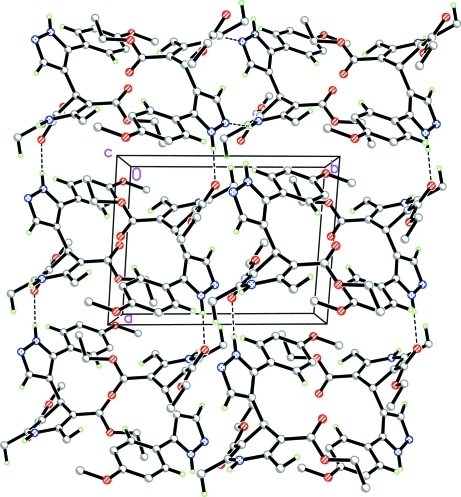
The crystal packing of the title compound, viewed along the c axis.

## supplementary crystallographic information

### Comment

Substituted pyridines are important structural components of a variety of biologically active compounds. They possess anti-inflammatory, anti-microbial (Surendra Kumar *et al.*, 2011), anti-oxidant, anti-tumor and anti-ulcer activities (Swarnalatha *et al.*, 2011). In view of these activities, herein we report the crystal structure of the title compound.

The molecular structure of the title compound is shown in Fig. 1. The pyrazole (N2/N3/C14–C16) ring is approximately planar with maximum deviation of  0.003 (1) Å for atom N2.  The central pyrazole (N2/N3/C14–C16) ring makes dihedral angles of 80.80 (6) and 38.16 (6)° with the pyridine (N1/C1–C5) ring and the benzene (C17–C22) ring, respectively.  The dihedral angle between the pyridine (N1/C1–C5) ring and the benzene (C17–C22) ring is 44.88 (5)°.

In the crystal packing, (Fig. 2), the adjacent molecules are connected *via* intra- and intermolecular N2—H1N2···O1, N1—H1N1···N3, C6—H6B···N3, C7—H7A···O3, C14—H14A···N1, C18—H18A···O1 and C22—H22A···O3 hydrogen bonds.

### Experimental

3-(4-Methoxyphenyl)-1*H*-pyrazole-4-carbaldehyde (0.2g, 0.80 mmol), ethylacetoacetate (0.26g, 1.6 mmol) and ammonium acetate (0.07g, 0.90 mmol) in ethanol (20 ml) were refluxed for 8 hours in an oil bath. After the completion of the reaction, the reaction mixture was concentrated and poured into crushed ice. The precipitated product was filtered and washed with water. The resulting solid was recrystallized from hot ethanol (yield: 0.32g, 76%; m.p. 453–455 K).

### Refinement

Atom H1N1 and H1N2 were located from a difference Fourier maps and refined freely [N—H = 0.880 (17)–0.906 (17) Å]. The remaining H atoms were positioned geometrically (C—H = 0.95–0.98 Å) and were refined using a riding model, with *U*_iso_(H) = 1.2 or 1.5*U*_eq_(C). A rotating group model was applied to the methyl groups.

### Crystal data

**Table:**

C_23_H_27_N_3_O_5_	*Z* = 2
*M**_r_* = 425.48	*F*(000) = 452
Triclinic, *P*1	*D*_x_ = 1.310 Mg m^−^^3^
Hall symbol: -P 1	Mo *K*α radiation, λ = 0.71073 Å
*a* = 8.5800 (1) Å	Cell parameters from 9992 reflections
*b* = 11.1286 (1) Å	θ = 2.4–32.9°
*c* = 11.4996 (1) Å	µ = 0.09 mm^−^^1^
α = 94.425 (1)°	*T* = 100 K
β = 99.191 (1)°	Block, yellow
γ = 92.992 (1)°	0.39 × 0.23 × 0.22 mm
*V* = 1078.37 (2) Å^3^	

### Data collection

**Table:**

Bruker SMART APEXII CCD area-detector diffractometer	7437 independent reflections
Radiation source: fine-focus sealed tube	5974 reflections with *I* > 2σ(*I*)
graphite	*R*_int_ = 0.022
φ and ω scans	θ_max_ = 32.0°, θ_min_ = 1.8°
Absorption correction: multi-scan (*SADABS*; Bruker, 2009)	*h* = −12→12
*T*_min_ = 0.965, *T*_max_ = 0.980	*k* = −16→16
28279 measured reflections	*l* = −17→17

### Refinement

**Table:**

Refinement on *F*^2^	Primary atom site location: structure-invariant direct methods
Least-squares matrix: full	Secondary atom site location: difference Fourier map
*R*[*F*^2^ > 2σ(*F*^2^)] = 0.046	Hydrogen site location: inferred from neighbouring sites
*wR*(*F*^2^) = 0.134	H atoms treated by a mixture of independent and constrained refinement
*S* = 1.05	*w* = 1/[σ^2^(*F*_o_^2^) + (0.0684*P*)^2^ + 0.3274*P*] where *P* = (*F*_o_^2^ + 2*F*_c_^2^)/3
7437 reflections	(Δ/σ)_max_ = 0.001
293 parameters	Δρ_max_ = 0.51 e Å^−^^3^
0 restraints	Δρ_min_ = −0.29 e Å^−^^3^

### Special details

**Table:**

Experimental. The crystal was placed in the cold stream of an Oxford Cryosystems Cobra open-flow nitrogen cryostat (Cosier & Glazer, 1986) operating at 100.0 (1) K.
Geometry. All s.u.'s (except the s.u. in the dihedral angle between two l.s. planes) are estimated using the full covariance matrix. The cell s.u.'s are taken into account individually in the estimation of s.u.'s in distances, angles and torsion angles; correlations between s.u.'s in cell parameters are only used when they are defined by crystal symmetry. An approximate (isotropic) treatment of cell s.u.'s is used for estimating s.u.'s involving l.s. planes.
Refinement. Refinement of F^2^ against ALL reflections. The weighted R-factor wR and goodness of fit S are based on F^2^, conventional R-factors R are based on F, with F set to zero for negative F^2^. The threshold expression of F^2^ > 2σ(F^2^) is used only for calculating R-factors(gt) etc. and is not relevant to the choice of reflections for refinement. R-factors based on F^2^ are statistically about twice as large as those based on F, and R- factors based on ALL data will be even larger.

### Fractional atomic coordinates and isotropic or equivalent isotropic displacement parameters (Å^2^)

**Table:**

	*x*	*y*	*z*	*U*_iso_*/*U*_eq_	
O1	0.16548 (9)	0.44561 (8)	0.87653 (7)	0.02135 (17)	
O2	0.38602 (9)	0.35073 (8)	0.93477 (7)	0.01969 (16)	
O3	0.47154 (11)	0.01240 (8)	0.66212 (9)	0.0295 (2)	
O4	0.25759 (10)	−0.00120 (7)	0.52149 (7)	0.02201 (17)	
O5	0.90976 (11)	0.04691 (9)	1.17677 (8)	0.0292 (2)	
N1	0.25821 (10)	0.36906 (8)	0.52613 (8)	0.01673 (17)	
N2	0.83784 (11)	0.41106 (8)	0.76648 (8)	0.01671 (17)	
N3	0.77514 (11)	0.47186 (9)	0.67396 (8)	0.01983 (18)	
C1	0.29133 (12)	0.24919 (10)	0.50498 (9)	0.01684 (19)	
C2	0.36451 (12)	0.19308 (9)	0.59735 (9)	0.01576 (18)	
C3	0.43164 (11)	0.26769 (9)	0.71308 (9)	0.01462 (18)	
H3A	0.4394	0.2133	0.7788	0.018*	
C4	0.31145 (12)	0.36043 (9)	0.73257 (9)	0.01510 (18)	
C5	0.24375 (12)	0.41694 (9)	0.63803 (9)	0.01594 (18)	
C6	0.15830 (13)	0.53096 (10)	0.64023 (10)	0.0202 (2)	
H6A	0.1846	0.5744	0.7190	0.030*	
H6B	0.0439	0.5110	0.6218	0.030*	
H6C	0.1905	0.5820	0.5813	0.030*	
C7	0.23910 (14)	0.19722 (11)	0.37946 (9)	0.0223 (2)	
H7A	0.3043	0.1305	0.3620	0.033*	
H7B	0.2510	0.2600	0.3257	0.033*	
H7C	0.1279	0.1672	0.3687	0.033*	
C8	0.37462 (13)	0.06117 (10)	0.59670 (9)	0.01829 (19)	
C9	0.25093 (15)	−0.13152 (10)	0.52548 (11)	0.0248 (2)	
H9A	0.2395	−0.1533	0.6057	0.030*	
H9B	0.3485	−0.1647	0.5043	0.030*	
C10	0.10914 (15)	−0.18010 (11)	0.43720 (11)	0.0263 (2)	
H10A	0.0967	−0.2680	0.4382	0.039*	
H10B	0.1238	−0.1600	0.3580	0.039*	
H10C	0.0143	−0.1440	0.4578	0.039*	
C11	0.27844 (12)	0.39157 (9)	0.85152 (9)	0.01611 (18)	
C12	0.36198 (14)	0.37357 (12)	1.05686 (10)	0.0241 (2)	
H12A	0.2559	0.3404	1.0665	0.029*	
H12B	0.3706	0.4614	1.0804	0.029*	
C13	0.48935 (16)	0.31170 (14)	1.13135 (11)	0.0306 (3)	
H13A	0.4835	0.3301	1.2152	0.046*	
H13B	0.5933	0.3406	1.1158	0.046*	
H13C	0.4741	0.2242	1.1115	0.046*	
C14	0.62754 (12)	0.42337 (10)	0.64436 (10)	0.0191 (2)	
H14A	0.5534	0.4484	0.5818	0.023*	
C15	0.59282 (12)	0.33141 (9)	0.71527 (9)	0.01463 (18)	
C16	0.73356 (12)	0.32572 (9)	0.79400 (9)	0.01473 (18)	
C17	0.77961 (12)	0.25038 (9)	0.89185 (9)	0.01571 (18)	
C18	0.87821 (13)	0.29968 (10)	0.99559 (9)	0.01787 (19)	
H18A	0.9163	0.3820	1.0023	0.021*	
C19	0.92046 (13)	0.22914 (11)	1.08843 (9)	0.0211 (2)	
H19A	0.9872	0.2634	1.1583	0.025*	
C20	0.86537 (13)	0.10797 (11)	1.07957 (10)	0.0216 (2)	
C21	0.77058 (14)	0.05728 (11)	0.97597 (10)	0.0229 (2)	
H21A	0.7349	−0.0256	0.9687	0.027*	
C22	0.72820 (13)	0.12868 (10)	0.88280 (10)	0.0202 (2)	
H22A	0.6634	0.0938	0.8123	0.024*	
C23	0.85276 (17)	−0.07678 (13)	1.16952 (13)	0.0332 (3)	
H23A	0.8849	−0.1095	1.2458	0.050*	
H23B	0.8972	−0.1230	1.1081	0.050*	
H23C	0.7371	−0.0827	1.1497	0.050*	
H1N2	0.941 (2)	0.4250 (14)	0.7995 (14)	0.027 (4)*	
H1N1	0.218 (2)	0.4093 (15)	0.4667 (15)	0.031 (4)*	

### Atomic displacement parameters (Å^2^)

**Table:**

	*U*^11^	*U*^22^	*U*^33^	*U*^12^	*U*^13^	*U*^23^
O1	0.0142 (4)	0.0257 (4)	0.0235 (4)	0.0027 (3)	0.0025 (3)	−0.0017 (3)
O2	0.0162 (4)	0.0277 (4)	0.0152 (3)	0.0038 (3)	0.0015 (3)	0.0024 (3)
O3	0.0270 (4)	0.0188 (4)	0.0375 (5)	0.0045 (3)	−0.0108 (4)	0.0004 (3)
O4	0.0196 (4)	0.0168 (4)	0.0268 (4)	−0.0008 (3)	−0.0033 (3)	0.0003 (3)
O5	0.0293 (5)	0.0338 (5)	0.0243 (4)	0.0007 (4)	−0.0021 (3)	0.0156 (4)
N1	0.0141 (4)	0.0181 (4)	0.0178 (4)	0.0012 (3)	0.0003 (3)	0.0051 (3)
N2	0.0121 (4)	0.0185 (4)	0.0191 (4)	0.0004 (3)	−0.0004 (3)	0.0056 (3)
N3	0.0141 (4)	0.0230 (4)	0.0225 (4)	0.0011 (3)	−0.0003 (3)	0.0096 (3)
C1	0.0130 (4)	0.0187 (5)	0.0182 (4)	−0.0013 (3)	0.0011 (3)	0.0019 (3)
C2	0.0130 (4)	0.0160 (4)	0.0177 (4)	−0.0001 (3)	0.0009 (3)	0.0010 (3)
C3	0.0120 (4)	0.0159 (4)	0.0158 (4)	0.0010 (3)	0.0012 (3)	0.0026 (3)
C4	0.0117 (4)	0.0158 (4)	0.0178 (4)	0.0005 (3)	0.0019 (3)	0.0026 (3)
C5	0.0109 (4)	0.0172 (4)	0.0196 (4)	−0.0002 (3)	0.0015 (3)	0.0033 (3)
C6	0.0165 (5)	0.0197 (5)	0.0249 (5)	0.0043 (4)	0.0020 (4)	0.0053 (4)
C7	0.0232 (5)	0.0254 (5)	0.0167 (4)	0.0007 (4)	−0.0012 (4)	0.0017 (4)
C8	0.0161 (5)	0.0174 (5)	0.0207 (5)	0.0002 (4)	0.0018 (4)	0.0002 (4)
C9	0.0244 (6)	0.0166 (5)	0.0314 (6)	0.0004 (4)	−0.0008 (4)	0.0011 (4)
C10	0.0231 (6)	0.0229 (5)	0.0307 (6)	−0.0012 (4)	0.0007 (4)	−0.0014 (4)
C11	0.0126 (4)	0.0160 (4)	0.0188 (4)	−0.0019 (3)	0.0006 (3)	0.0011 (3)
C12	0.0201 (5)	0.0360 (6)	0.0162 (5)	0.0006 (4)	0.0038 (4)	0.0005 (4)
C13	0.0261 (6)	0.0466 (8)	0.0188 (5)	0.0018 (5)	0.0006 (4)	0.0064 (5)
C14	0.0134 (4)	0.0225 (5)	0.0214 (5)	0.0011 (4)	−0.0003 (4)	0.0088 (4)
C15	0.0120 (4)	0.0156 (4)	0.0161 (4)	0.0009 (3)	0.0009 (3)	0.0030 (3)
C16	0.0135 (4)	0.0150 (4)	0.0156 (4)	0.0017 (3)	0.0014 (3)	0.0028 (3)
C17	0.0134 (4)	0.0174 (4)	0.0162 (4)	0.0020 (3)	0.0010 (3)	0.0031 (3)
C18	0.0162 (5)	0.0189 (5)	0.0178 (4)	0.0010 (4)	0.0004 (3)	0.0020 (4)
C19	0.0186 (5)	0.0264 (5)	0.0169 (4)	0.0009 (4)	−0.0019 (4)	0.0037 (4)
C20	0.0184 (5)	0.0268 (5)	0.0203 (5)	0.0026 (4)	0.0012 (4)	0.0103 (4)
C21	0.0212 (5)	0.0198 (5)	0.0263 (5)	−0.0013 (4)	−0.0023 (4)	0.0080 (4)
C22	0.0188 (5)	0.0194 (5)	0.0206 (5)	0.0001 (4)	−0.0030 (4)	0.0042 (4)
C23	0.0304 (6)	0.0343 (7)	0.0372 (7)	0.0016 (5)	0.0040 (5)	0.0219 (6)

### Geometric parameters (Å, °)

**Table:**

O1—C11	1.2275 (13)	C7—H7C	0.9800
O2—C11	1.3430 (12)	C9—C10	1.5017 (17)
O2—C12	1.4565 (13)	C9—H9A	0.9900
O3—C8	1.2093 (13)	C9—H9B	0.9900
O4—C8	1.3398 (13)	C10—H10A	0.9800
O4—C9	1.4530 (14)	C10—H10B	0.9800
O5—C20	1.3655 (13)	C10—H10C	0.9800
O5—C23	1.4282 (17)	C12—C13	1.5075 (17)
N1—C5	1.3810 (14)	C12—H12A	0.9900
N1—C1	1.3896 (14)	C12—H12B	0.9900
N1—H1N1	0.880 (17)	C13—H13A	0.9800
N2—N3	1.3584 (12)	C13—H13B	0.9800
N2—C16	1.3604 (13)	C13—H13C	0.9800
N2—H1N2	0.906 (17)	C14—C15	1.4056 (14)
N3—C14	1.3317 (14)	C14—H14A	0.9500
C1—C2	1.3574 (14)	C15—C16	1.3963 (14)
C1—C7	1.5021 (15)	C16—C17	1.4708 (13)
C2—C8	1.4746 (14)	C17—C22	1.3931 (15)
C2—C3	1.5254 (14)	C17—C18	1.4029 (14)
C3—C15	1.5165 (14)	C18—C19	1.3867 (14)
C3—C4	1.5258 (14)	C18—H18A	0.9500
C3—H3A	1.0000	C19—C20	1.3960 (17)
C4—C5	1.3627 (13)	C19—H19A	0.9500
C4—C11	1.4602 (14)	C20—C21	1.3921 (16)
C5—C6	1.4988 (15)	C21—C22	1.3959 (14)
C6—H6A	0.9800	C21—H21A	0.9500
C6—H6B	0.9800	C22—H22A	0.9500
C6—H6C	0.9800	C23—H23A	0.9800
C7—H7A	0.9800	C23—H23B	0.9800
C7—H7B	0.9800	C23—H23C	0.9800
			
C11—O2—C12	116.61 (8)	C9—C10—H10C	109.5
C8—O4—C9	115.93 (9)	H10A—C10—H10C	109.5
C20—O5—C23	116.58 (10)	H10B—C10—H10C	109.5
C5—N1—C1	121.27 (9)	O1—C11—O2	121.98 (10)
C5—N1—H1N1	116.6 (11)	O1—C11—C4	126.11 (9)
C1—N1—H1N1	119.6 (11)	O2—C11—C4	111.89 (9)
N3—N2—C16	112.67 (8)	O2—C12—C13	106.44 (10)
N3—N2—H1N2	120.6 (10)	O2—C12—H12A	110.4
C16—N2—H1N2	126.6 (10)	C13—C12—H12A	110.4
C14—N3—N2	103.98 (8)	O2—C12—H12B	110.4
C2—C1—N1	117.80 (9)	C13—C12—H12B	110.4
C2—C1—C7	127.83 (10)	H12A—C12—H12B	108.6
N1—C1—C7	114.37 (9)	C12—C13—H13A	109.5
C1—C2—C8	124.03 (9)	C12—C13—H13B	109.5
C1—C2—C3	119.30 (9)	H13A—C13—H13B	109.5
C8—C2—C3	116.54 (8)	C12—C13—H13C	109.5
C15—C3—C2	114.71 (8)	H13A—C13—H13C	109.5
C15—C3—C4	109.82 (8)	H13B—C13—H13C	109.5
C2—C3—C4	106.30 (8)	N3—C14—C15	113.01 (9)
C15—C3—H3A	108.6	N3—C14—H14A	123.5
C2—C3—H3A	108.6	C15—C14—H14A	123.5
C4—C3—H3A	108.6	C16—C15—C14	103.80 (9)
C5—C4—C11	121.66 (9)	C16—C15—C3	129.74 (9)
C5—C4—C3	118.34 (9)	C14—C15—C3	126.03 (9)
C11—C4—C3	119.91 (8)	N2—C16—C15	106.53 (9)
C4—C5—N1	118.28 (9)	N2—C16—C17	120.75 (9)
C4—C5—C6	127.35 (10)	C15—C16—C17	132.71 (9)
N1—C5—C6	114.32 (9)	C22—C17—C18	118.77 (9)
C5—C6—H6A	109.5	C22—C17—C16	120.81 (9)
C5—C6—H6B	109.5	C18—C17—C16	120.41 (9)
H6A—C6—H6B	109.5	C19—C18—C17	120.51 (10)
C5—C6—H6C	109.5	C19—C18—H18A	119.7
H6A—C6—H6C	109.5	C17—C18—H18A	119.7
H6B—C6—H6C	109.5	C18—C19—C20	120.28 (10)
C1—C7—H7A	109.5	C18—C19—H19A	119.9
C1—C7—H7B	109.5	C20—C19—H19A	119.9
H7A—C7—H7B	109.5	O5—C20—C21	124.32 (11)
C1—C7—H7C	109.5	O5—C20—C19	115.95 (10)
H7A—C7—H7C	109.5	C21—C20—C19	119.73 (10)
H7B—C7—H7C	109.5	C20—C21—C22	119.77 (10)
O3—C8—O4	122.38 (10)	C20—C21—H21A	120.1
O3—C8—C2	124.30 (10)	C22—C21—H21A	120.1
O4—C8—C2	113.15 (9)	C17—C22—C21	120.91 (10)
O4—C9—C10	106.00 (9)	C17—C22—H22A	119.5
O4—C9—H9A	110.5	C21—C22—H22A	119.5
C10—C9—H9A	110.5	O5—C23—H23A	109.5
O4—C9—H9B	110.5	O5—C23—H23B	109.5
C10—C9—H9B	110.5	H23A—C23—H23B	109.5
H9A—C9—H9B	108.7	O5—C23—H23C	109.5
C9—C10—H10A	109.5	H23A—C23—H23C	109.5
C9—C10—H10B	109.5	H23B—C23—H23C	109.5
H10A—C10—H10B	109.5		
			
C16—N2—N3—C14	0.59 (12)	C5—C4—C11—O2	−163.27 (9)
C5—N1—C1—C2	−23.57 (14)	C3—C4—C11—O2	13.14 (13)
C5—N1—C1—C7	156.10 (10)	C11—O2—C12—C13	175.86 (10)
N1—C1—C2—C8	165.32 (10)	N2—N3—C14—C15	−0.41 (13)
C7—C1—C2—C8	−14.30 (17)	N3—C14—C15—C16	0.10 (13)
N1—C1—C2—C3	−10.26 (14)	N3—C14—C15—C3	173.13 (10)
C7—C1—C2—C3	170.12 (10)	C2—C3—C15—C16	−124.23 (11)
C1—C2—C3—C15	−81.50 (12)	C4—C3—C15—C16	116.16 (11)
C8—C2—C3—C15	102.59 (10)	C2—C3—C15—C14	64.59 (14)
C1—C2—C3—C4	40.06 (12)	C4—C3—C15—C14	−55.03 (13)
C8—C2—C3—C4	−135.85 (9)	N3—N2—C16—C15	−0.54 (12)
C15—C3—C4—C5	82.46 (11)	N3—N2—C16—C17	−179.99 (9)
C2—C3—C4—C5	−42.18 (12)	C14—C15—C16—N2	0.26 (11)
C15—C3—C4—C11	−94.07 (11)	C3—C15—C16—N2	−172.41 (10)
C2—C3—C4—C11	141.30 (9)	C14—C15—C16—C17	179.61 (11)
C11—C4—C5—N1	−168.89 (9)	C3—C15—C16—C17	6.94 (19)
C3—C4—C5—N1	14.65 (14)	N2—C16—C17—C22	−141.80 (11)
C11—C4—C5—C6	13.84 (16)	C15—C16—C17—C22	38.92 (17)
C3—C4—C5—C6	−162.62 (10)	N2—C16—C17—C18	37.31 (15)
C1—N1—C5—C4	21.24 (14)	C15—C16—C17—C18	−141.96 (12)
C1—N1—C5—C6	−161.14 (9)	C22—C17—C18—C19	−1.53 (16)
C9—O4—C8—O3	1.75 (17)	C16—C17—C18—C19	179.34 (10)
C9—O4—C8—C2	−173.77 (9)	C17—C18—C19—C20	0.08 (17)
C1—C2—C8—O3	159.34 (12)	C23—O5—C20—C21	−0.38 (18)
C3—C2—C8—O3	−24.97 (16)	C23—O5—C20—C19	179.30 (11)
C1—C2—C8—O4	−25.24 (15)	C18—C19—C20—O5	−178.22 (10)
C3—C2—C8—O4	150.45 (9)	C18—C19—C20—C21	1.47 (18)
C8—O4—C9—C10	178.33 (10)	O5—C20—C21—C22	178.13 (11)
C12—O2—C11—O1	0.11 (15)	C19—C20—C21—C22	−1.54 (18)
C12—O2—C11—C4	−178.22 (9)	C18—C17—C22—C21	1.46 (17)
C5—C4—C11—O1	18.49 (17)	C16—C17—C22—C21	−179.41 (10)
C3—C4—C11—O1	−165.10 (10)	C20—C21—C22—C17	0.06 (18)

### Hydrogen-bond geometry (Å, °)

**Table:**

*D*—H···*A*	*D*—H	H···*A*	*D*···*A*	*D*—H···*A*
N2—H1N2···O1^i^	0.906 (17)	1.981 (17)	2.8858 (12)	176.3 (14)
N1—H1N1···N3^ii^	0.880 (17)	2.173 (17)	2.9969 (13)	155.7 (16)
C6—H6B···N3^iii^	0.98	2.50	3.4076 (15)	154
C7—H7A···O3^iv^	0.98	2.59	3.5561 (15)	167
C14—H14A···N1	0.95	2.60	3.2484 (14)	126
C18—H18A···O1^v^	0.95	2.48	3.1594 (14)	128
C22—H22A···O3	0.95	2.28	3.2210 (15)	170

Symmetry codes: (i) *x*+1, *y*, *z*; (ii) −*x*+1, −*y*+1, −*z*+1; (iii) *x*−1, *y*, *z*; (iv) −*x*+1, −*y*, −*z*+1; (v) −*x*+1, −*y*+1, −*z*+2.
